# Deubiquitinases and the new therapeutic opportunities offered to cancer

**DOI:** 10.1530/ERC-14-0516

**Published:** 2015-02

**Authors:** Roland Pfoh, Ira Kay Lacdao, Vivian Saridakis

**Affiliations:** Department of Biology, York University, 4700 Keele Street, Toronto, Ontario, Canada, M3J1P3

**Keywords:** deubiquitinases, USP7, USP22, CYLD, UCHL1, BAP1, A20, ataxin 3

## Abstract

Deubiquitinases (DUBs) play important roles and therefore are potential drug targets in various diseases including cancer and neurodegeneration. In this review, we recapitulate structure–function studies of the most studied DUBs including USP7, USP22, CYLD, UCHL1, BAP1, A20, as well as ataxin 3 and connect them to regulatory mechanisms and their growing protein interaction networks. We then describe DUBs that have been associated with endocrine carcinogenesis with a focus on prostate, ovarian, and thyroid cancer, pheochromocytoma, and adrenocortical carcinoma. The goal is enhancing our understanding of the connection between dysregulated DUBs and cancer to permit the design of therapeutics and to establish biomarkers that could be used in diagnosis and prognosis.

## Ubiquitination

The ubiquitin–proteasome system (UPS) has many critical regulatory roles in eukaryotic cellular processes including cell cycle progression, stress response, signal transduction, transcriptional activation, and DNA repair (Ciechanover *et al*. 2000, Ciechanover 2006). Malfunction, dysregulation, or molecular defects within the UPS lead to several different disease states including various cancers as well as neurodegenerative disorders (Ciechanover 2003, Ciechanover & Schwartz 2004). The UPS is responsible for the regulation, stability, and turnover of many different substrate proteins. However, the post-translational ubiquitination of a target protein has many other fates within eukaryotic cells as well.

Ubiquitin is a 76-residue eukaryotic polypeptide that is covalently attached to target proteins through an isopeptide bond formed between its terminal Gly residue and the ε-amino group of a lysine residue on the target protein. The ubiquitin superfamily of proteins possesses a similar three-dimensional structure, called the ββαββ fold or β-grasp fold, in the absence of sequence similarity. Proteins other than ubiquitin that possess this fold are called ubiquitin-like proteins and ubiquitin-like domains are found in many different proteins involved in the ubiquitination system. Ubiquitin is encoded by four different genes (*UBB*, *UBC*, *UBA52*, and *UBA80* (*RPS27A*)) and is produced as a precursor that must be proteolytically processed to release mature ubiquitin. The ubiquitin amino acid sequence is much conserved between eukaryotic organisms with only two residue changes between the yeast and human proteins. A single ubiquitin moiety can be attached to substrate proteins resulting in mono-ubiquitination. Mono-ubiquitination is involved in a variety of cellular processing including protein translocation, kinase activation, epigenetic regulation, and DNA damage signaling (Miranda & Sorkin 2007, Wang *et al*. 2012). The N-terminal methionine and the seven lysine residues in ubiquitin (K6, K11, K27, K29, K33 K48, and K63) can form polyubiquitin chains. The attachment of a polyubiquitin chain to substrate proteins results in a variety of cellular effects. The best characterized is Lys48 (K48) polyubiquitination, which targets proteins for degradation by the 26S proteasome complex (Hochstrasser 1996, Hicke 2001). Lys63 (K63) polyubiquitination plays important roles in DNA damage signaling and recruitment of DNA repair proteins to the site of damage (Panier & Durocher 2009). K63 and linear polyubiquitination function in the activation of the nuclear factor kappa-light-chain-enhancer of activated B cells (NFκB) signaling pathway (Chen 2005, Terzic *et al*. 2007, Iwai & Tokunaga 2009). Ubiquitination plays many additional roles depending on the chain configuration and linkage (Komander 2009). There is increasing evidence for the formation of more complex topologies with mixed linkages with unknown functions.

**Figure 1:**
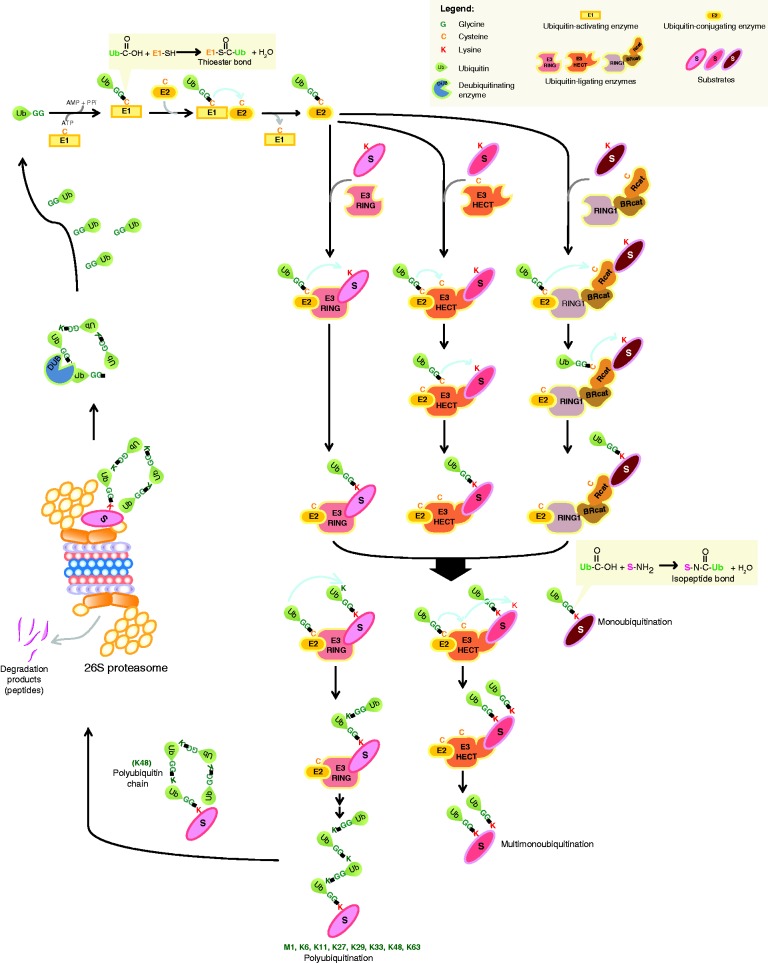
Enzymatic cascades resulting in the ubiquitination, deubiquitination, and 26S proteasome degradation of substrate proteins.

The attachment of ubiquitin to target proteins is mediated by an enzymatic cascade consisting of E1 (ubiquitin-activating enzyme), E2 (ubiquitin-conjugating enzymes), and E3 (ubiquitin-protein ligases) proteins (Fig. 1; Fang & Weissman 2004). E1 is the ubiquitin-activating enzyme that catalyzes the ATP-dependent activation of ubiquitin resulting in the formation of a thioester bond between the C-terminus of ubiquitin and a cysteine residue in the E1 active site. Activated ubiquitin is subsequently transferred to an E2 ubiquitin-conjugating enzyme and again forms a thioester bond between its C-terminus and a cysteine residue in the E2 active site. There are at least four different classes of E3 ubiquitin protein ligases whose function is to recognize and catalyze the ubiquitination of the substrate protein (Pickart 2001). The first class of E3 ubiquitin protein ligases contains a Really Interesting New Gene (RING) domain and acts as a scaffold between the ubiquitin-conjugated E2 and the target protein to be ubiquitinated. The RING domain-containing protein is essential for target protein recognition and mediates the transfer of the ubiquitin from the E2 onto the target protein. Another class of E3 ligases contains a homologous to the E6-AP carboxyl terminus (HECT) domain. Unlike RING domain proteins, HECT domain proteins actually catalyze the transfer of ubiquitin from the loaded E2 onto a cysteine residue in their active site forming a thioester bond and then finally onto the substrate protein. The third class is called U-box, whose members function like RING E3 ligases and have a RING-like fold; however, they lack the cysteine and histidine Zn-coordination sites (Aravind & Koonin 2000). The latest identified class of E3 ligases is the RING-inbetween-RING (RBR) family, which are hybrid RING/HECT type E3 ligases containing three variant RING domains, known as RING1, IBR/BRcat, and RING2/Rcat (Smit & Sixma 2014, Spratt *et al*. 2014). Similar to HECT proteins, they catalyze the transfer of ubiquitin to a cysteine in the active site of the RING2/Rcat domain. The RING1 domain mediates interactions with the E2 enzyme. The specificity and type of ubiquitination is generated from substrate recognition by the E3 and the formation of different E2–E3 complex combinations that mediate ubiquitination (Weissman 2001).

## Deubiquitination

**Figure 2:**
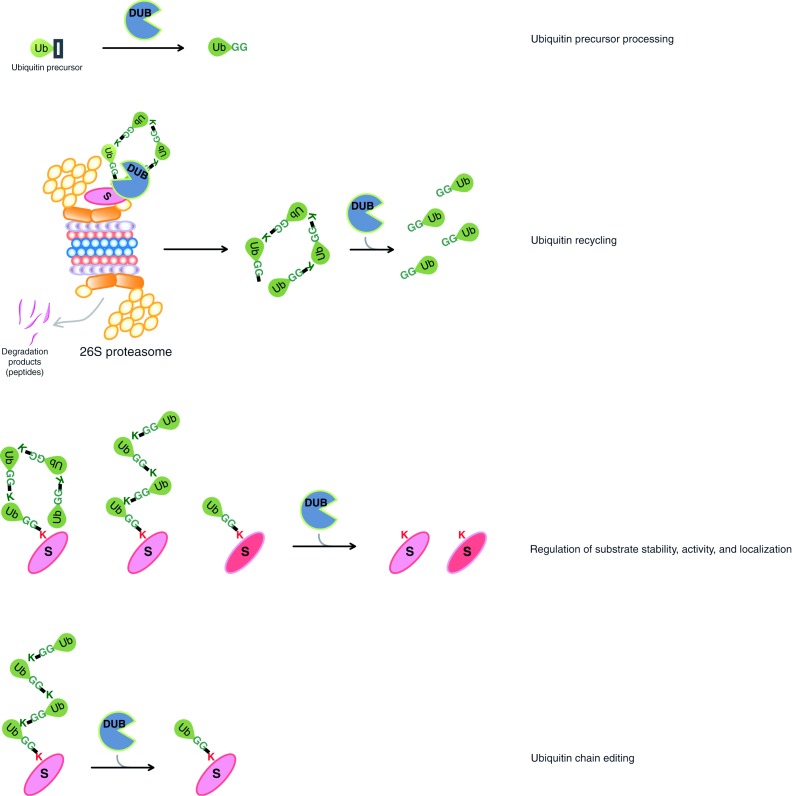
Reactions catalyzed by deubiquitinases (DUBs). (A) Mature ubiquitin is generated following DUB cleavage. (B) DUBs recycle ubiquitin on proteins destined for degradation by the 26S proteasome. (C) DUBs deubiquitinate substrate proteins. (D) DUBs regulate ubiquitin chain editing.

Protein ubiquitination is both dynamic and reversible. Deubiquitinases or deubiquitinating enzymes (DUBs) catalyze the removal of ubiquitin from target proteins and are also involved in ubiquitin maturation, recycling, and editing (Fig. 2; Kim *et al*. 2003, Amerik & Hochstrasser 2004, Nijman *et al*. 2005, Reyes-Turcu & Wilkinson 2009, Clague *et al*. 2013). Processes that can be regulated by deubiquitination include the rescue of proteins that are destined for degradation, the cleavage to release mature ubiquitin, the editing of a polyubiquitin on a substrate protein as well as the removal of ubiquitin to terminate or alter a biological event. DUBs are associated with the 26S proteasome to rescue ubiquitin chains before the degradation of the substrate protein. These unanchored polyubiquitin chains are then processed by other DUBs to replenish the free ubiquitin pool in the cell. DUBs are divided into two main classes according to their enzymatic cleavage mechanism: cysteine proteases and zinc metalloproteases. The larger group of cysteine proteases is further divided into four classes according to sequence conservation in the catalytic domain. Currently, the five known DUB families include ubiquitin C-terminal hydrolases (UCHs), ubiquitin-specific proteases (USPs), Machado–Joseph disease (MJD) protein domain proteases, ovarian tumor proteases (OTUs), and JAMM motif zinc metalloproteases (Nijman *et al*. 2005). There are ∼90 human DUBs; however, the biological roles and target proteins are unknown for many of them (Nijman *et al*. 2005, Clague *et al*. 2013). The importance of DUBs in cellular functions as well as carcinogenesis has been recently reviewed (Luise *et al*. 2011, D'Arcy & Linder 2012, Fraile *et al*. 2012, Bhattacharya & Ghosh 2014*a*, Pal *et al*. 2014). By far, USPs are the largest class of DUBs with ∼60 proteases in humans, their sizes ranging from ∼50–300 kDa and most of them containing several domains in addition to the catalytic domain. Sequence conservation among these proteases is limited to the catalytic domain, which is characterized by an active site catalytic triad containing Cys, His, and Asp (or Asn) residues. As the non-catalytic domains are highly diverse at the amino acid sequence level, it is hypothesized that they are important for conferring substrate specificity, regulation of the catalytic activity, and mediating protein interactions to the individual USPs. UCHs were the first identified family of DUBs with four members in humans. The role of UCHs was initially proposed to be in the salvage and maturation of ubiquitin, as they are unable to cleave long polyubiquitin chains. The remaining families of DUBs, OTU, Josephin, and JAMM are described later in this review. DUB specificity and regulation are currently being investigated. With so many DUBs, it is easy to imagine that each DUB will have different modes of regulation and substrates. Most importantly, the majority of DUBs are found in protein complexes, whereby specificity and regulation can be controlled. Other DUBs are activated by post-translational modifications including phosphorylation, sumoylation, and ubiquitination. Some DUBs are specific for their target substrates and some DUBs are also specific for the ubiquitin chain type (K48 vs K63 linked). Most DUBs specifically recognize ubiquitin and cannot catalyze the proteolysis of ubiquitin-like proteins. DUBs also have specificity that determines where proteolysis occurs with some DUBs cleaving from the end of a polyubiquitin chain, whereas others cleave internally.

## Deubiquitinases

All DUBs that play important roles in pathways that are dysregulated in cancer including DNA repair, cell growth, and apoptosis are potential drug targets. However, the difficulty in development of effective drugs may be the challenge in designing specific inhibitors for a single DUB. This challenge is similar to that encountered in the design of inhibitors against protein kinases that have hampered drug development for many years. However, there are currently several potent kinase inhibitor drugs in the market, indicating that these concerns are indeed valid and challenging but certainly not insurmountable. The three-dimensional structures of the catalytic domains of USP2, USP7, USP8, and USP14 have revealed striking structural conservation of their active site, thus substantiating the concerns that drug development may be difficult (Hu *et al*. 2002, 2005, Avvakumov *et al*. 2006, Renatus *et al*. 2006). Interestingly, these crystal structures also revealed that these catalytic domains were in inactive conformations before ubiquitin binding, suggesting alternative targets for drug design. As the binding of ubiquitin activates USPs, it is desirable to identify compounds that bind to the catalytic domains and prevent the binding of ubiquitin. However, in parallel to targeting the catalytic domains of USPs for drug design, it would be advantageous to target individual substrate recognition, regulatory or protein interaction domains, as this would avoid concerns about cross-reactivity. Each USP has at least one or more of these domains and there is no sequence or structure conservation between most USP domains making these attractive drug-interaction sites. The ability to prevent complex formation between USPs and their ubiquitinated substrates is desirable for the design of drug compounds that interfere with specific deubiquitination processes. Therefore, structural analysis to reveal the molecular mechanism of interaction between USPs and their substrates will greatly enhance the development of these inhibitors. However, directly targeting large protein–protein interaction surfaces is quite challenging and requires precise structural knowledge of the interaction sites. The ability to understand the pathways and proteins that are involved in key regulatory events leading to disease will allow the development of therapeutics that targets only these proteins.

The structure–function relationships of selected DUBs will be discussed in the following section. We chose at least one example from each of the five DUB classes (USP-class: USP7, CYLD, and USP22; OTU-class: A20; UCH-class: UCHL1 and BRCA1-associated protein 1 (BAP1); MJD-class: ataxin 3 (ATXN3); and JAMM-class: Rpn11). Within their classes, the DUBS were selected based on the number of publications, disease relevance, as well as the available data on structure–function relationships. Overall, USP7, CYLD, UCHL1, BAP1, A20, and ATXN3 are the most intensively studied DUBS based on the number of citations in PubMed. All of the selected DUBS are related to cancer.

## Ubiquitin-specific protease 7

USP7 was initially identified through its interaction with the ICP0 protein of herpes simplex virus (Meredith *et al*. 1994). Subsequently, USP7 was also shown to interact with the EBNA1 protein from Epstein–Barr virus, vIRF4 and LANA proteins from Kaposi's sarcoma herpesvirus, UL35 from cytomegalovirus, and E1B-55K from adenovirus (Holowaty *et al*. 2003, Saridakis *et al*. 2005, Lee *et al*. 2011, Jager *et al*. 2012, Salsman *et al*. 2012, Ching *et al*. 2013). Therefore, USP7 has emerged as a common target of herpesviruses (and perhaps other DNA viruses) living up to its original name of herpesvirus-associated USP (HAUSP).

USP7 regulates the turnover of many critical proteins involved in tumor suppression and DNA repair. p53, a tumor suppressor that induces cell cycle arrest and apoptosis, was the first protein to be identified as a USP7 substrate (Li *et al*. 2002*a*). Apart from p53, USP7 also deubiquitinates and stabilizes Hdm2 and HdmX, which are negative regulators of p53, indicating a complex regulatory mechanism (Meulmeester *et al*. 2005). Hdm2 is preferentially targeted by USP7 over p53 under normal unstressed conditions; however, USP7 targets p53 in response to DNA damage (Meulmeester *et al*. 2005). It has recently been shown that both GMPS and Abro1 modulate USP7-mediated deubiquitination of p53 after DNA damage (Reddy *et al*. 2014, Zhang *et al*. 2014). The deletion of the *USP7* gene resulted in early embryonic lethality partly due to increased p53 levels (Kon *et al*. 2010). USP7 regulates the localization, rather than the stability, of tumor suppressors phosphatase and tensin homolog (PTEN) and FOXO4 by deubiquitinating their monoubiquitinated, transcriptionally active, nuclear forms (van der Horst *et al*. 2006, Song *et al*. 2008). USP7 also plays important roles in genomic stability by deubiquitinating and stabilizing many proteins involved in these pathways including BUB3 (Giovinazzi *et al*. 2014), claspin (Faustrup *et al*. 2009), ARF-BP1 (Khoronenkova & Dianov 2013), and others. These USP7 substrate proteins play critical roles in pathways that are often dysregulated in cancer highlighting the importance of investigating USP7 as a therapeutic target in cancers involving these proteins.

GMPS is not only involved in nucleotide biosynthesis but also modulates the function of USP7 deubiquitination of both H2B and p53, resulting in epigenetic silencing and DNA repair respectively (van der Knaap *et al*. 2005, 2009, Sarkari *et al*. 2009, Reddy *et al*. 2014). The role of USP7 in epigenetic silencing is not limited to H2B deubiquitination, as it also deubiquitinates and stabilizes RING1B, BMI1, UHRF1, and DNMT1, proteins that are involved in chromatin remodeling and epigenetic regulation (de Bie *et al*. 2010, Du *et al*. 2010, Maertens *et al*. 2010).

**Figure 3:**
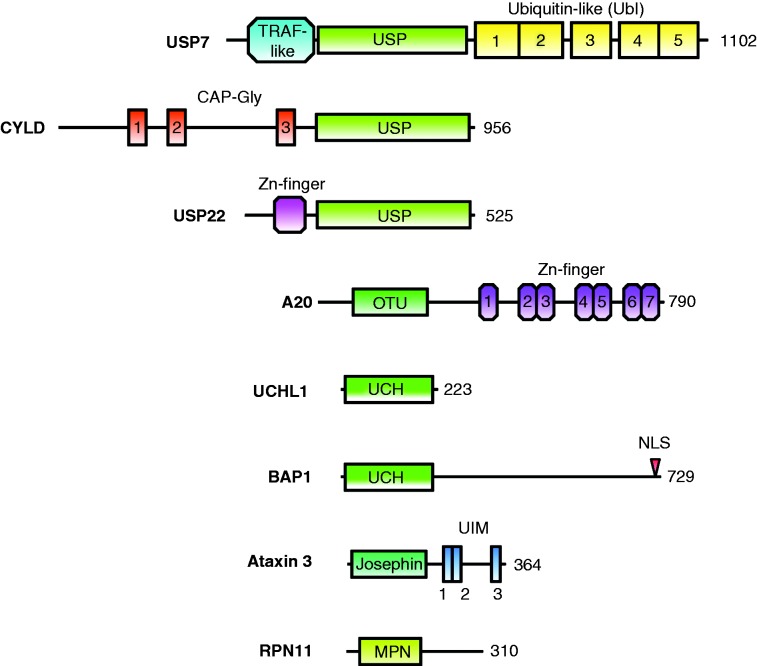
Domain architecture of selected DUBs: USP7, CYLD, USP22, A20, UCHL1, BAP1, ataxin 3, and RPN11.

Individual USP7 domains have been structurally characterized, including the catalytic domain with bound ubiquitin-aldehyde (Hu *et al*. 2002), the TNF receptor-associated factor (TRAF)-like N-terminal domain (USP7-NTD) with various peptides from different substrates (p53, Hdm2, HdmX, and UbE2E1) (Sheng *et al*. 2006, Sarkari *et al*. 2010, 2013) as well as the five ubiquitin-like domains of the C-terminus (Faesen *et al*. 2011) (Fig. 3). USP7-NTD is a protein–protein interaction domain involved primarily in substrate recognition, whereas USP7-CTD is involved in protein–protein interactions and regulation of catalytic activity.

The ability to understand how and when USP7 deubiquitinates each substrate is essential to our overall understanding of its function. Owing to its regulation of Hdm2, HdmX, and p53, USP7 is an exemplary USP drug target. In cancers that are caused by a dysregulated Hdm2 protein but retain WT p53, the ability to inhibit USP7 deubiquitination of Hdm2 is considered to be of therapeutic importance because Hdm2 degradation would lead to p53 stabilization and activation. Therefore, a drug that prevents the formation of the Hdm2/USP7 complex or inhibits its catalytic activity will benefit patients with cancer associated with a dysregulated Hdm2 protein. Elevated levels of USP7 have been recently discovered in prostate cancer and glioma (Song *et al*. 2008, Bhattacharya & Ghosh 2014*b*). USP7 was also implicated to modulate tumor growth and apoptosis in a colon carcinoma xenograft model (Becker *et al*. 2008). As well, reduced expression of USP7 was observed in non-small cell lung carcinomas (Masuya *et al*. 2006). The recent discoveries of new USP7 substrates (such as Rb and BMI) implicate its possible involvement in other cancers indicating a need for USP7 therapeutics (Maertens *et al*. 2010, Bhattacharya & Ghosh 2014*b*).

Several USP7 inhibitors have been tested in a variety of *in vitro* and *in vivo* assays (Colland *et al*. 2009, Chauhan *et al*. 2012, Reverdy *et al*. 2012, Weinstock *et al*. 2012, Fan *et al*. 2013, Yamaguchi *et al*. 2013). HBX 41,108 and P5091 are USP7 inhibitors with IC_50_ values of submicromolar concentrations (Colland *et al*. 2009, Chauhan *et al*. 2012). The potency of HBX 41,108 has been studied in HCT116, human colon carcinoma cells, where it was shown to inhibit cell growth and induce apoptosis (Colland *et al*. 2009). The potency of P5091 was shown to induce apoptosis in multiple myeloma cells resistant to bortezomib (Chauhan *et al*. 2012). However, to date, there have not been any studies evaluating USP7 inhibitors in endocrine-related cancers.

## CYLD

Similar to USP7, CYLD belongs to the USP class of cysteine proteases (Fig. 3). Together with A20 (see below), CYLD regulates the pathway of NFκB (Brummelkamp *et al*. 2003, Trompouki *et al*. 2003), which is a key regulatory protein in the response of the immune system to infections. Dysregulation of the NFκB pathway is involved not only in cancer (Massoumi 2011), but also in a number of other diseases such as viral infection (Chan & Greene 2011) and inflammatory diseases (Harhaj & Dixit 2012). It has been recently suggested that CYLD down-regulation could increase breast cancer metastasis through NFκB activation (Hayashi *et al*. 2014). The USP domain of CYLD is located at the C-terminus and contains a zinc-binding B-box domain. Though similar to the RING finger, the zinc-binding domain does not show E3 ligase activity and does not alter enzymatic activity or substrate specificity, but is relevant for the localization of CYLD to the cytoplasm (Komander *et al*. 2008). Compared with other USP domains (e.g. USP7), the USP finger region involved in ubiquitin binding is shorter, which could explain the preference of CYLD for K63-linked ubiquitin chains (Komander *et al*. 2008). In addition to the USP domain, CYLD contains three cytoskeleton-associated protein-glycine-conserved (CAP-Gly) domains. Its three-dimensional fold is similar to the SH domain, which is a binding motif for proline-rich sequences (Saito *et al*. 2004). CYLD negatively regulates the NFκB pathway by deubiquitinating critical factors such as NFκB essential modulator (NEMO, also called IKKγ) and TRAF2 (Kovalenko *et al*. 2003).

## Ubiquitin-specific protease 22

USP22 consists of 525 amino acids and contains an N-terminal UBP-type zinc finger and a C-terminal USP domain. It is involved in the deubiquitination of NAD-dependent protein deacetylase sirtuin 1 (SIRT1; Lin *et al*. 2012), as well as histones H2A and H2B, as part of the transcriptional regulatory histone acetylation and deubiquitination complex known as Spt-Ada-Gcn5 acetyltransferase (SAGA). Structural data on USP22 are not available, but its yeast ortholog Ubp8 (ubiquitin processing protease 8) has been crystallized within the deubiquitination module of the SAGA complex (Köhler *et al*. 2010, Samara *et al*. 2010). The SAGA DUB module is a tight complex between Ubp8, Sgf11, Sgf73, and Sus1, and Ubp8 is allosterically regulated by its binding partners (Samara *et al*. 2012). USP22 is involved in oncogenesis due to its role in a large variety of cancers (Schrecengost *et al*. 2014). USP22 expression is increased in laryngeal squamous cell carcinoma (Li *et al*. 2014), salivary adenoid cystic carcinoma (Dai *et al*. 2014), cervical cancer (Yang *et al*. 2014), salivary duct carcinoma (Piao *et al*. 2013), colorectal carcinoma (Liu *et al*. 2010), breast cancer (Zhang *et al*. 2011*a*), oral squamous cell carcinoma (Piao *et al*. 2012), and papillary carcinoma (Wang *et al*. 2013). The overexpression of USP22 is related to poor prognosis and may be used as a biomarker during diagnosis. USP22 has been identified as a cancer stem cell marker in the ‘death-from-cancer’ signature genes study (Glinsky 2005, 2006, Glinsky *et al*. 2005). Genome-wide expression-profiling analysis identified 11 genes that could be used as markers to predict both metastases and failure of cancer treatment.

## A20

By far, the most intensely studied member of the OTU-class of cysteine proteases is A20 (alternative name TNFAIP3: tumor necrosis factor alpha-induced protein); together with CYLD, it negatively regulates the NFκB pathway (Fig. 3). A20 is regulated by estrogen and A20 levels are highly increased in aggressive forms of breast cancer and involved in tamoxifen resistance (Vendrell *et al*. 2007), suggesting a potential role both as a marker and as a therapeutic target. However, A20 levels are decreased in B-cell lymphomas, leading to NFκB activation and contributing to their resistance to apoptosis (Hymowitz & Wertz 2010). Unlike most other DUBs, A20 also possesses an E3 ubiquitin ligase function. This dual E3 ligase–DUB function allows A20 to elegantly inhibit NFκB-related signaling by removing K63-linked polyubiquitin chains from substrate molecules such as TRAF6 and receptor-interacting serine/threonine protein kinase 1 (RIP1) and label them for 26S proteasomal degradation by attaching a K48-linked polyubiquitin chain (Wertz *et al*. 2004). A20 is generated in response to NFκB-induced gene activation (Krikos *et al*. 1992) and is thus part of the negative feedback mechanism in the NFκB pathway. For distinct functions in the NFκB pathway, A20 requires other proteins such as tax1-binding protein 1 (TAX1BP1), E3 ubiquitin-protein ligase Itchy homolog (ITCH), and RING finger protein 11 (RNF11) to form an A20 ubiquitin-editing complex (Shembade *et al*. 2011). The C-terminus of A20 contains seven zinc finger (ZnF) domains, of which ZnF4 is essential for E3 ligase activity (Wertz *et al*. 2004). Furthermore, this domain binds ubiquitin (Bosanac *et al*. 2010, Tokunaga *et al*. 2012) and TAX1BP1 (Verstrepen *et al*. 2011). The ZnF1 domain is essential for recognition of RIP1 (Bosanac *et al*. 2010). ZnF4 has been crystallized with mono-ubiquitin and shows a high affinity for K63-linked polyubiquitin chains (Bosanac *et al*. 2010), whereas ZnF7 crystallized with linear di-ubiquitin (Tokunaga *et al*. 2012). A comparison between the crystal structures of the N-terminal OTU-domain and otubain 2 (OTUB2) revealed different structural features around the catalytic triad, suggesting differences in substrate specificity as well as catalytic activity of the individual DUBs (Nanao *et al*. 2004, Komander & Barford 2008, Lin *et al*. 2008). Although the core architecture of the catalytic triad was found to be very similar in the USP- and OTU-domains, A20 showed variations in the third catalytic residue. A more recent crystallographic study has reported reversible oxidation of the catalytic cysteine residue in A20 and other OTU–DUBs (Kulathu *et al*. 2013), which is stabilized by the specific nature of the catalytic center and has been suggested as a regulatory mechanism.

## UCHL1 (PGP 9.5)

UCHL1 (also known as PGP 9.5) is the most studied member of the UCH-class of cysteine protease DUBs and is associated with multiple types of cancers including lung (Hibi *et al*. 1998), colorectal (Yamazaki *et al*. 2002), pancreatic (Leiblich *et al*. 2007), and breast (Xiang *et al*. 2012) cancers as well as neurodegenerative diseases such as Parkinson's (Leroy *et al*. 1998) and Alzheimer's (Xue & Jia 2006) (Fig. 3). It is a marker for neuroendocrine cells and tumors (Thompson *et al*. 1983, Rode *et al*. 1985) and has been suggested as a detection tool for pancreatic (Kato *et al*. 2013, Tomita 2013), parathyroid (Howell *et al*. 2009), ovarian (Brait *et al*. 2013), and breast cancers (Liu *et al*. 2014). UCHL1 cleaves single amino acids or small peptides from the C-terminus of ubiquitin precursors to create mono-ubiquitin, but does not cleave ubiquitin from proteins (Larsen *et al*. 1998). Dimeric UCHL1 displays E3 ubiquitin ligase activity *in vitro* (Liu *et al*. 2002). With only 223 amino acids, it is among the shortest DUBs, containing only a UCH domain. UCHL1 adopts a papain-like α–β–α fold (Das *et al*. 2006) similar to UCHL3 and UCHL5. The crystal structure of apo-UCHL1 showed a distorted catalytic triad (Das *et al*. 2006), whereas with bound ubiquitin vinyl methyl ester the catalytic triad realigns into an active form (Boudreaux *et al*. 2010). A similar scenario is present in USP7, where the catalytic triad was only properly aligned with bound ubiquitin-aldehyde (Hu *et al*. 2002). A tripeptide fluoromethylketone inhibitor has been shown to bind at the active site to the misaligned UCHL1 form (Davies *et al*. 2012). The precise physiological role of UCHL1 is somewhat elusive. In healthy subjects, it is exclusively expressed in the neurons and testis, but it is found in cancerous tissues. Mutations in UCHL1 have been linked to Parkinson's disease (Leroy *et al*. 1998). Both protein and mRNA levels of UCHL1 are down-regulated in prostate cancer due to hypo-methylation of the *UCHL1* promoter (Ummanni *et al*. 2011).

## BRCA1-associated protein 1

Research into BAP1 has gained attention due to its role in lung and breast cancers (Jensen *et al*. 1998) and tumor suppression (Ventii *et al*. 2008) (Fig. 3). An extensive study found mutations and deletions to *BAP1* to be particularly associated with the detrimental outcome of various common cancers, especially in renal clear cell carcinoma (Kandoth *et al*. 2013). BAP1 is named according to its interaction with the RING finger domain of BRCA1, which is an ubiquitin E3 ligase involved in DNA repair. Although BRCA1 itself is not a substrate of BAP1, its function is affected by BAP1. A recent study has linked the deubiquitination of γ-tubulin by BAP1 to breast cancer (Zarrizi *et al*. 2014). *Drosophila* BAP1 has been found to form the so-called polycomb repressive DUB (PR-DUB) complex with the polycomb group (PcG) protein ASX (additional sex combs protein), which deubiquitinates mono-ubiquitinated histone H2A (Scheuermann *et al*. 2010). The corresponding PR-DUB complex in humans involves BAP1 and host cell factor 1 (HCFC1). It had been observed earlier that HCFC1 is a substrate of BAP1, suggesting that BAP1 regulates cell proliferation (Machida *et al*. 2009, Misaghi *et al*. 2009). A recent study has found that the transcription factor forkhead box protein K2 (FOXK2) guides the PR-DUB complex to its targets on the genome via BAP1 binding (Ji *et al*. 2014). There is no structural information for BAP1. In addition to its UCH domain, BAP1 contains a long C-terminal extension. The C-terminal extension contains a functional nuclear core localization (NLS) domain and several binding sites for interaction partners such as BRCA1 and HCFC1. For tumor suppression, both the enzymatic activity and the NLS domain are essential (Ventii *et al*. 2008).

## Ataxin 3

ATXN3 belongs to the MJD class of cysteine proteases and is named after spinocerebellar ataxia type 3, a neurodegenerative disease, which is also called MJD (Fig. 3). A recent study has reported that ATXN3 represses the transcription of *PTEN* (Sacco *et al*. 2014), an immensely important tumor suppressor (Alimonti *et al*. 2010) critically linked to endocrine-related cancers (Fu *et al*. 2014). The regulation of PTEN by ATXN3 highlights an earlier study that suggested involvement of ATXN3 in gene expression by binding to specific DNA sequences as well as histone deacetylase 3 (HDAC3) and nuclear receptor corepressor (NCoR) (Evert *et al*. 2006). In addition to the N-terminal catalytic Josephin domain, ATXN3 contains two C-terminal ubiquitin-interaction motifs (UIMs), which are also found in the 26S proteasome (Hofmann & Falquet 2001). ATXN3 shows preferred binding and cleavage of longer poly-ubiquitin, with a minimal chain length of four (Burnett *et al*. 2003). Neighboring the two UIMs is a poly-glutamine (polyQ) stretch. ATXN3 mutations with extended polyQ stretches cause MJD (Gusella & MacDonald 2000). Ironically, normal ATXN3 is involved in the regulation of protein aggregates and supposedly plays a role in the suppression of polyQ-related neurodegenerative diseases such as Huntington's disease (Warrick *et al*. 2005). Critical for this suppressive function are the UIM domains, the catalytic activity as well as proteasome activity. It has been suggested that the deubiquitinating function of ATXN3 protects against its own intrinsic polyQ toxicity (Warrick *et al*. 2005). Binding of ATXN3 to valosin-containing protein (VCP, also called p97) suggested a regulatory involvement of ATXN3 in endoplasmatic reticulum-associated degradation (Zhong & Pittman 2006), which is a quality control system targeting misfolded proteins for proteasomal digestion. ATXN3 regulates C-terminus of Hsc70-interacting protein (Scaglione *et al*. 2011), which is an E3 ligase that collaborates with chaperones in protein quality control. The catalytic Josephin domain of ATXN3 investigated by solution NMR (Mao *et al*. 2005, Nicastro *et al*. 2005) revealed structural similarity to the catalytic domains of other cysteine proteases and ubiquitin binding.

## Rpn11

Rpn11 (also known as PSMD14 or POH1) is an essential enzyme of the 26S proteasome and belongs to the 19S regulatory particle non-ATPase (Rpn) subunits (Fig. 3). Rpn11 is located in the lid of the proteasome in a hetero-dimeric complex with Rpn8 (Lander *et al*. 2012). It cleaves K48-linked ubiquitin chains from proteasome substrates, which is necessary for proteasomal processing and degradation (Yao & Cohen 2002). Only recently, the involvement of the proteasome in DNA repair has been reported (Ben-Aroya *et al*. 2010). In this respect, Rpn11 has been found to promote the double-strand DNA break response by restricting K63-linked ubiquitin chains attached to histones near damaged DNA sites (Butler *et al*. 2012). It had been observed earlier that Rpn11 can specifically cleave K63-linked poly-ubiquitin chains (Cooper *et al*. 2009), which is contrary to its cleavage of K48-linked chains targeted for proteasomal digestion, but fits to the K63 specificity of other zinc metalloproteases such as AMSH (associated molecule with the SH3 domain of signal transducting adapter molecule 1) and AMSH-LP (AMSH-like protein) (Sato *et al*. 2008). Rpn11 has also been connected to drug resistance in cancer (Spataro *et al*. 2002) as well as idiopathic nephrotic syndrome (Ma *et al*. 2009), which is a common kidney disease in children. Rpn11 alone is not active (Yao & Cohen 2002), and in complex with Rpn8 it displays low activity (Pathare *et al*. 2014), suggesting that it is only fully functional when embedded into the 26S proteasome.

## Endocrine cancers

The remainder of this review focuses on DUBs that have already been implicated in endocrine cancers.

### Prostate cancer

Prostate cancer is the second most common cancer diagnosed and the second most common cause of cancer death in men worldwide. In the USA, an estimated 233 000 new cases of prostate cancer were predicted to be diagnosed in 2014 and ∼30 000 associated deaths (Siegel *et al*. 2014). Prostate cancer is more frequently diagnosed in men over the age of 65. Screening for prostate cancer is based on the prostate-specific antigen blood test; however, abnormal levels require prostate biopsies for definitive diagnosis as well as digital rectal examination. Combinations of tumor surveillance, surgery, radiation, hormonal, and chemotherapies are used in the management and treatment of prostate cancer. The molecular basis of disease is quite complex and mutations in many different oncogenes and tumor suppressor genes have been implicated including *TP53*, *RB1*, *EZH2*, *BMI*, and *PTEN* (Wang & Wong 1997, Dean & Knudsen 2013, Yang & Yu 2013). All of these genes have also been implicated in most other cancers; therefore, none are directly linked to prostate cancer alone. In light of all of the identified mutations, prostate cancer appears to be caused by dysregulation of many different cellular signaling pathways especially those involved in cell survival and apoptosis.

Interestingly, the DUB USP7 has been implicated in the regulation of several of these genes including *TP53*, *RB1*, *BMI*, and *PTEN* (Li *et al*. 2002*a*, Song *et al*. 2008, Maertens *et al*. 2010, Bhattacharya & Ghosh 2014*b*). The involvement of overexpressed USP7 has already been described in prostate cancer via dysregulation of PTEN (Song *et al*. 2008). PTEN is a lipid and protein phosphatase that catalyzes the dephosphorylation of phosphatidylinositol (3,4,5)-triphosphate inhibiting the Akt/PKB signaling pathway. However, PTEN also has nuclear functions, whereby it regulates chromatin and DNA repair processes. It has recently become clear that in many cancers, PTEN is excluded from the nucleus (Planchon *et al*. 2008). The ubiquitination of PTEN is one mechanism that leads to its nuclear translocation. In the nucleus, USP7 catalyzes the deubiquitination of PTEN leading to its export from the nucleus (Song *et al*. 2008). This USP7-dependent deubiquitination and removal of PTEN from the nucleus is associated with many cancers (Song *et al*. 2008). More recently, the ability of USP7 to deubiquitinate PTEN was found to be under the control of BCR-ABL (Morotti *et al*. 2014). BCR-ABL phosphorylates USP7 and activates the deubiquitination of PTEN. A USP7 inhibitor could target prostate cancers; however, the effectiveness of such an inhibitor needs further study. Single nucleotide polymorphisms in Hdm2, HdmX, and USP7 have also been associated with aggressive prostate cancer (Sun *et al*. 2010). The involvement of USP7 in other mechanisms of prostate cancer awaits further investigations.

USP2a (also known as USP2) has been implicated in prostate cancer through its deubiquitination and stabilization of fatty acid synthase (Graner *et al*. 2004) and more recently Hdm2 (Stevenson *et al*. 2007) as well as its closely related functional homolog HdmX (Allende-Vega *et al*. 2010). Furthermore, overexpressed USP2a can transform prostate epithelial cells *in vitro* and NIH3T3 cells overexpressing USP2a caused the growth of tumors in all 12 injected nude mice *in vivo* (Priolo *et al*. 2006). More importantly, USP2a was found to be overexpressed in prostate cancer and is regulated by androgens in a concentration-dependent manner (Graner *et al*. 2004). The inactivation of USP2a via knockdown, mutations, or low expression has a negative effect on fatty acid synthase levels and p53-regulated genes, which enhances apoptosis (Graner *et al*. 2004, Priolo *et al*. 2006). USP2a levels were found to be up-regulated along with fatty acid synthase-related genes in almost half of all prostate cancers. These studies suggested an important role for USP2a in prostate cancer. It is therefore expected that an USP2a inhibitor could have important therapeutic applications. The c-myc oncogene is overexpressed in a subset of USP2a overexpressing prostate cancer cells (Benassi *et al*. 2012). The stabilization of Hdm2 by USP2a results in enhanced degradation of p53, thus preventing apoptosis. This enhanced p53 degradation has a direct consequence in MYC up-regulation by repressing the expression of regulatory microRNAs that target the *MYC* mRNA. The derepression of microRNAs miR-34b/c, miR-98, and let-7c resulting in increased levels of MYC is attributed to increased levels of USP2a (Benassi *et al*. 2012). Inhibitors targeting the catalytic activity of USP2a could attack prostate cancer cells via several different mechanisms, such as i) reducing MYC levels, thus reducing proliferation and ii) increasing p53 levels leading to increased apoptosis.

USP19 silencing directly affected the growth of several prostate cancer cell lines, suggesting a putative role in carcinogenesis (Lu *et al*. 2011). USP19 deubiquitinates and stabilizes KPC1, an E3 ligase for p27. Interestingly, the effects of decreased nuclear levels of p27, resulting in poor prognosis, have already been described in prostate cancer (Chu *et al*. 2008). USP19 regulates the levels of p27, although p27 is not a USP19 substrate.

One of the functions of USP26 is to regulate the activity and stability of the androgen receptor in the nucleus (Dirac & Bernards 2010). The androgen receptor is a steroid hormone receptor that is responsible for male development and maturation. It is activated by binding androgens such as testosterone or dihydrotestosterone and is transported to the nucleus where it functions as a transcription factor. The androgen receptor has been implicated in several cancers including testicular (Garolla *et al*. 2005), prostate (Heinlein & Chang 2004), breast (Cochrane *et al*. 2014), and thyroid cancers (Stanley *et al*. 2012). Investigation of the effects of USP26 in prostate cancer is necessary given its role in activity and stability of the androgen receptor and the role of the androgen receptor in prostate cancer (Wosnitzer *et al*. 2014). Similar to USP26, USP12 catalyzes the deubiquitination and stabilization of the androgen receptor (Burska *et al*. 2013). Furthermore, the functional role of USP12 in the increased transcriptional activation of the androgen receptor has been extended to the Akt pathway through the pro-apoptotic phosphatases (PHLPP and PHLPPL). The USP12/Uaf-1/WDR20 deubiquitinating complex also stabilizes both PHLPP and PHLPPL eventually resulting in increased transcriptional activation by the androgen receptor (Burska *et al*. 2013). Even though the role of USP10 in prostate cancer has not been investigated, it is interesting to note that USP10 is also involved in the regulation of the androgen receptor (Draker *et al*. 2011).

Betulinic acid, derived from the bark of white birch trees, induces apoptosis quite selectively in a variety of cancer cells (Gheorgheosu *et al*. 2014). Betulinic acid had already been described to inhibit prostate cancer growth though a mechanism of action involving the decreased levels of VEGF and survivin due to the increased ubiquitin-mediated degradation of specificity protein transcription factors (Sp1, Sp3, and Sp4; Chintharlapalli *et al*. 2007). Another study determined that betulinic acid increases the levels of poly-ubiquitinated proteins and their degradation and concluded that betulinic acid most probably inhibits the actions of several as yet unidentified DUBs (Reiner *et al*. 2013).

### Ovarian cancer

In the USA, an estimated 22 000 new cases of ovarian cancer were predicted to be diagnosed in 2014 resulting in ∼14 000 deaths associated with this disease (Siegel *et al*. 2014). Ovarian cancer is asymptomatic until later stages making it quite difficult to diagnose. The number of deaths is quite high because of the late stages of disease and metastases. The greatest risk factor for ovarian cancer is genetic with several common mutations. The current understanding of the involvement of DUBs in ovarian cancer is rather limited. Many tumor suppressor genes including *TP53*, *PTEN*, *RB1*, and *BRCA1* are either mutated or dysregulated in ovarian cancer. Therefore, as USP7 regulates all of these proteins, the role of USP7 in ovarian cancer needs to be investigated. The ubiquitin carboxyl terminal hydrolases UCH37 (also known as UCHL5) and UCHL1 have both been implicated in ovarian cancer. As in other cancers, UCH37 has been found to be up-regulated and linked to poor prognosis (Wang *et al*. 2014). UCHL1 has been identified as being both up-regulated (Jin *et al*. 2013) and down-regulated in ovarian cancer (Okochi-Takada *et al*. 2006, Jin *et al*. 2013). *UCHL1* knockdown in ovarian cancer cell lines where it was overexpressed caused increased proliferation. Another study that set out to identify both up- and down-regulated genes in ovarian cancer for use in diagnosis determined that USP36 was overexpressed (Li *et al*. 2008). More importantly, USP36 was identified in sera of 36% of cases from ovarian cancer patients compared with none from healthy subjects. Further investigations are needed to define the role of USP36 in ovarian and other cancers and confirm its ability to be useful in diagnosis. USP15 is amplified in ovarian and breast cancers as well as glioblastoma (Eichhorn *et al*. 2012). USP15 regulates the TGFβ and NFκB signaling pathways by deubiquitinating the type 1 TGFβ receptor as well as IκBα (Schweitzer *et al*. 2007, Eichhorn *et al*. 2012). Proteins from both of these pathways have already been implicated as possible cancer therapeutic targets. Down-regulation of USP15 has been observed in paclitaxel-resistant ovarian cancer (Xu *et al*. 2009). Furthermore, knockdown of USP15 in HeLa cells also lead to paclitaxel resistance corroborating its important role in this resistance. Krüppel-like factor 8 (KLF8) is a transcription factor that is highly overexpressed in ovarian and other cancers. KLF8 can transform ovarian epithelial cells alone and in combination with other oncogenes by down-regulating USP44 (Lu *et al*. 2014). USP44 plays a role in cancer through its dysregulation of the DNA damage response mediated by the E3 ligase RNF168 on histone H2A (Mosbech *et al*. 2013). USP44 is a tumor suppressor protein that is silenced in a variety of other cancers (Holland & Cleveland 2012, Sloane *et al*. 2014).

### Pheochromocytoma

Pheochromocytoma is a usually benign cancer originating in the adrenal glands that is associated with Von Hippel–Lindau disease, neurofibromatosis, and multiple endocrine neoplasia type 2 and characterized by excessive secretion of catecholamines (Tsirlin *et al*. 2014). Von Hippel–Landau disease is associated with various other malignancies as well (Tootee & Hasani-Ranjbar 2012). The following discussion continues only with Von Hippel–Landau disease, as it is related to the ubiquitin system. Von Hippel–Lindau disease is caused by mutations in the gene encoding the VHL protein (pVHL). The VHL protein associates with elongin B, elongin C, and cullin 2 to form an E3 ligase complex that is responsible for the regulation of hypoxia inducible factor 1 alpha (HIF1α). Under normoxia, pVHL catalyzes the poly-ubiquitination and degradation of HIF1α by interacting with a 2-hydroxylated proline on HIF1α. However, under conditions of hypoxia, HIF1α is not hydroxylated, therefore pVHL is unable to interact and catalyze its poly-ubiquitination. As a transcription factor, HIF1α induces expression of genes containing hypoxia response elements that are involved in angiogenesis, glucose metabolism, and invasion/metastasis (Li *et al*. 2005). Mutations in pVHL disrupt the ubiquitination of HIF1α. Dysregulated degradation of HIF1α results in enhanced expression of its target genes. Two DUBs, VDU1 and VDU2 (pVHL-interacting deubiquitinating enzymes 1 and 2), are also known substrates of the pVHL E3 ligase complex (Li *et al*. 2002*b*). VDU1 is also known as USP33 and VDU2 as USP20. Both of these DUBs share sequence and functional similarities and are ubiquitinated by the pVHL–E3 ligase complex and targeted for degradation (Li *et al*. 2002*b*). In addition, VDU2, but not VDU1, is able to interact with and deubiquitinate HIF1α, and rescue it from proteasome-mediated degradation. Interestingly, pVHL regulates the levels of both HIF1α and VDU2 leading to a vital equilibrium between the degradation and rescue of HIF1α. The inability of the pVHL–E3 ligase complex to catalyze the ubiquitination of not only HIF1α but also VDU1 and VDU2 may lead to disease. As DUBs are substrates of the pVHL–E3 ligase complex, it is important to note that both of these DUBs are emerging targets in the development of therapeutics against Von Hippel–Landau disease. The design of inhibitors against VDU1 and VDU2 could prevent non-regulated deubiquitination of substrate proteins to compensate for the inability of the pVHL–E3 ligase complex to degrade it. USP20 is involved in the deubiquitination and rescue of RAD17, a protein involved in the DNA damage response pathway (Shanmugam *et al*. 2014). The knockdown of *USP20* caused the sensitization of two different cancer cell lines to cisplatin (Shanmugam *et al*. 2014), strongly suggesting that an inhibitor against USP20 may function as a cancer therapeutic drug.

### Thyroid cancer

In the USA, an estimated 63 000 new cases of thyroid cancer were predicted to be diagnosed in 2014 resulting in ∼1800 deaths (Siegel *et al*. 2014). USP22 is overexpressed in papillary carcinoma, a type of thyroid cancer (Wang *et al*. 2013). Increased USP22 protein levels were shown for papillary carcinoma tissue when compared with noncancerous tissue using immunohistochemistry (Wang *et al*. 2013). Kaplan–Meier analysis indicated that the survival of patients with papillary carcinoma was significantly lower with high USP22 expression levels. Taken together, the authors concluded that USP22 could be functioning as an oncogene and used in the prognosis of papillary carcinoma. There is one case of MALT lymphoma of the thyroid that has been associated with the deletion of *A20* (Chanudet *et al*. 2009). The expression of OTUD1 is highly up-regulated in various carcinomas of the thyroid and could potentially be used as a biomarker to distinguish between cancerous samples (Carneiro *et al*. 2014).

UCHL1 belongs to the ubiquitin-carboxyl terminal hydrolases family of DUBs. This family of proteases catalyzes the hydrolysis of ubiquitin from small adducts to maintain cellular ubiquitin homeostasis. It is the main DUB responsible for maintaining cellular levels of monomeric ubiquitin primarily by hydrolyzing ester and amide forms of ubiquitin. It is highly expressed in neuronal and endocrine cells; however, its expression has been detected in many non-neuronal cancers. The role of UCHL1 in endocrine cancers is well established. The up-regulation of UCHL1 has been observed in one of the six cases of granular cell tumors located in the thyroid gland (Espinosa-de-Los-Monteros-Franco *et al*. 2009). UCHL1 is also known as PGP9.5, a well-established marker for diagnosis and prognosis of a variety of cancers. PGP9.5 was evaluated as a prognostic marker in invasive colorectal, pancreatic, parathyroid, and lung cancers (Hibi *et al*. 1999, Tezel *et al*. 2000, Yamazaki *et al*. 2002, Howell *et al*. 2009). Patients with pancreatic cancer that were positive for PGP9.5 had lower survival rates than those who were negative for PGP9.5 (Tezel *et al*. 2000). There were significant differences in maximal tumor size and the extent of tumor in patients with invasive colorectal cancer expressing PGP9.5 (Yamazaki *et al*. 2002). PGP9.5 was evaluated as a diagnostic marker in medullary thyroid carcinoma (Takano *et al*. 2004). Overexpression of *PGP9.5* mRNA was identified in all eleven medullary thyroid carcinoma samples examined. This study showed that levels of *PGP9.5* mRNA were similar to normal thyroid tissues in other thyroid cancers including anaplastic, papillary, and follicular carcinomas as well as follicular adenoma, suggesting that overexpression of PGP9.5 could not be used as a biomarker for these cancers.

Both VDU1 (USP33) and VDU2 (USP20) also play important biological roles related to the thyroid. VDU1 and VDU2 deubiquitinate and thus reactivate the hormone-activating type 2 deiodinase (D2), which is an endoplasmic reticulum integral membrane protein (Curcio-Morelli *et al*. 2003, Gereben *et al*. 2008). D2 functions in the conversion of the inactive precursor thyroxine, into triiodothyroxine (T_3_), the active hormone responsible for cellular energy and metabolism homeostasis. Therefore, very tight control of D2 levels is critical. The mechanism by which levels of T_3_ are controlled involves the ubiquitination leading to inactivation and the subsequent degradation of D2. D2 is ubiquitinated by the WSB-1 and TEB4 E3 ligases in response to D2 activation and increased levels of T_3_ (Dentice *et al*. 2005, Zavacki *et al*. 2009). However, the process of D2 degradation can be reversed by VDU1- and VDU2-catalyzed deubiquitination, resulting in D2 rescue and reactivation. It is unknown whether the deubiquitination of D2 has any roles in VHL disease or cancer (Curcio-Morelli *et al*. 2003).

### Adrenocortical carcinoma

Adrenocortical adenoma and carcinoma are tumors of the adrenal cortex. Adrenocortical carcinoma is a rare but very aggressive cancer with a 5-year survival rate of 30%. Adenomas on the other hand are benign tumors. The up-regulation of USP4 and USP38 was identified in adrenocortical carcinoma using microarray gene expression analysis (Laurell *et al*. 2009). USP4 had previously been identified as being up-regulated in adrenocortical carcinoma using transcriptional profiling (Velazquez-Fernandez *et al*. 2005). Several USP4 deubiquitinating targets have been identified including ARF-BP1, type 1 TGFβ receptor, and PDK1 (Zhang *et al*. 2011*b*, 2012, Uras *et al*. 2012). The roles of ARF-BP1 and PDK1 in adrenocortical carcinomas have not yet been investigated. The TGF signaling pathway has been implicated in the tumorigenicity of adrenocortical carcinomas (Yamamoto *et al*. 2006, Parviainen *et al*. 2013).

## Therapeutic targeting of DUBs for the treatment of cancer

The studies thus far describing the involvement of DUBs in endocrine cancers have only scratched the surface of the many roles that DUBs may play in these cancers (Table 1). Additional studies are needed to define the actual landscape of DUBs that are mutated, deleted, or differentially regulated in these cancers. These studies, along with the design of targeted, small molecule DUB inhibitors, will most probably lead to new opportunities for endocrine cancer therapeutics and diagnostics.

DUBs have been implicated in several types of cancers and neurodegenerative diseases. As such, targeting DUBs can lead to effective therapies in the treatment, diagnosis, and prevention of these diseases. The UPS regulates many important proteins; therefore, it is not surprising that, when UPS components are acting aberrantly, accelerated degradation or mislocalization of substrate proteins results in disease. Various studies have suggested the dual roles of DUBs, both as oncogenes and as tumor suppressors. With DUBs having multiple substrates, the nature of their substrates may distinguish their oncogenic vs suppressive roles.

Velcade (bortezomib) was the first clinically approved UPS inhibitor and is used as a therapeutic agent for B cell lymphoma and multiple myeloma, exemplifying the importance of the UPS as an effective therapeutic target (Bold 2004). Kyprolis (carfilzomib), another proteasome inhibitor, was FDA approved in 2012 for multiple myeloma (Steele 2013). The biggest challenge in therapeutics is that a proteasome inhibitor is non-specific and indeed bortezomib and carfilzomib treatments are quite toxic. A potentially more attractive therapeutic approach would be inhibitors designed with increased specificity against DUBs.

## Concluding remarks

A more comprehensive understanding of the roles, substrates, and regulation of DUBs will lead to a better understanding of their emerging roles in carcinogenesis and clinical applications of their inhibitors. The continued and accelerated development of small molecule inhibitors against DUBs will lead to increased success in the treatment of various cancers and other diseases.

## Figures and Tables

**Table 1:** DUBs in endocrine cancers

**Cancer**	**DUBs**	**References**
Prostate	USP2	Graner *et al*. (2004), Priolo *et al*. (2006) and Benassi *et al*. (2012)
	USP7	Song *et al*. (2008)
	USP10	Draker *et al*. (2011)
	USP12	Leiblich *et al*. (2007) and Burska *et al*. (2013)
	USP14	Du *et al*. (2015)
	USP19	Lu *et al*. (2011)
	USP22	Schrecengost *et al*. (2014)
	USP26	Dirac & Bernards (2010)
	USP39	Wen *et al*. (2014)
	UCHL1	Leiblich *et al*. (2007)
Ovarian	USP2a	Yang *et al*. (2007)
	USP36	Li *et al*. (2008)
	USP44	Lu *et al*. (2014)
	OTUD1	Carneiro *et al*. (2014)
Thyroid	USP20	Curcio-Morelli *et al*. (2003) and Li *et al*. (2005)
	USP22	Wang *et al*. (2013)
	USP33	Li *et al*. (2002*c*) and Curcio-Morelli *et al*. (2003)
	A20	Chanudet *et al*. (2009)
	UCHL1	Takano *et al*. (2004)
Adrenal cortex	USP4	Laurell *et al*. (2009)
	USP38	Laurell *et al*. (2009)
Parathyroid	UCHL1	Howell *et al*. (2009)
